# Sensorial and Nutritional Aspects of Cultured Meat in Comparison to Traditional Meat: Much to Be Inferred

**DOI:** 10.3389/fnut.2020.00035

**Published:** 2020-03-24

**Authors:** Ilse Fraeye, Marie Kratka, Herman Vandenburgh, Lieven Thorrez

**Affiliations:** ^1^Research Group for Technology and Quality of Animal Products, Leuven Food Science and Nutrition Research Centre, KU Leuven Ghent Technology Campus, Gent, Belgium; ^2^Department of Development and Regeneration, KU Leuven, Kortrijk, Belgium; ^3^Department of Pathology, Brown University, Providence, RI, United States

**Keywords:** post-mortem metabolism, texture, flavor, color, nutritional composition, cultivated meat, clean meat

## Abstract

Cultured meat aspires to be biologically equivalent to traditional meat. If cultured meat is to be consumed, sensorial (texture, color, flavor) and nutritional characteristics are of utmost importance. This paper compares cultured meat to traditional meat from a tissue engineering and meat technological point of view, focusing on several molecular, technological and sensorial attributes. We outline the challenges and future steps to be taken for cultured meat to mimic traditional meat as closely as possible.

## Introduction

In 2013, the first cultured meat prototype in the shape of a hamburger was presented in the media (1). The hamburger was based on 10,000 strips containing myotubes engineered in a hydrogel. However, the engineered muscle-like tissues also required the addition of colorants (beetroot juice), flavors (saffron and caramel), and texturizers (bread crumbs and a binder) to make the patty similar in appearance to a hamburger (2). Producing a high-quality hamburger from traditional meat does not require the addition of these ingredients, suggesting that the intrinsic characteristics of the cultured cells differed significantly from traditional meat. The tasting panel commented that the burger tasted a little dry due to a lack of fat, but no profound quality or sensorial assessment was performed. The only other, modest, sensorial test on cultured cells reported in scientific literature, dates back to the early years of cultured meat experimentation and included smelling and observation, but no tasting (3). In addition, several review papers briefly discussed the potential sensorial characteristics of cultured meat (or derived products) (4, 5), but most of the information provided was based on indirect assumptions and on knowledge of the current *in vitro* production capabilities. To our knowledge, a scientific and technological comparison between cultured meat and traditional meat has not been published thus far. This relates to the fact that cultured meat is currently not available in sufficient quantities to conduct, such assessments. Still, based on the currently available state of the art concerning the production process of cultured meat, important considerations with regard to the technological, sensorial and nutritional characteristics of cultured meat can be inferred.

Cultured meat aspires to be biologically equivalent to traditional meat (6). If cultured meat is to be consumed, sensorial characteristics (texture, color, flavor) are of utmost importance. These sensorial properties are derived from the molecular characteristics of the product, such as the content and nature of the proteins, the presence of myoglobin, the composition of volatile compounds, etc. In addition to sensorial attributes, the nutritional quality of cultured meat should also resemble its traditional counterpart as closely as possible. Traditional meat is a nutritionally dense food containing high-quality proteins, vitamins, minerals, and other important nutrients (7, 8). It is of interest to note that many compounds that accumulate in the muscle are not produced in the muscle but derive from animal feed components which have been digested and modified by non-muscle organs. Unless specifically added to the culture medium and taken up by the cells, these compounds would be absent in cultured meat, influencing processes determining flavor, texture, color and nutritional aspects.

## Post-Mortem Metabolism

When a farm animal is slaughtered, muscles are transformed into meat through a complex biochemical process. The lack of oxygen supply results in a metabolic shift toward anaerobic glycolysis, by which glycogen present in the muscle cell is converted to lactate. This results in an intracellular pH drop from around 7 (in the living animal) to ~5.4–5.8. Due to calcium release from the sarcoplasmic reticulum, muscle contraction is initiated. As the ATP concentration in the cell drops, muscle contraction ceases at a state in which actin and myosin heads closely interact (*rigor mortis*), forming the permanent actomyosin complex (7, 9, 10). This muscle contraction and complex formation significantly influence the properties of the meat. On the one hand, tenderness and water holding capacity decrease (9). The formation of the actomyosin complex necessitates the use of phosphate, releasing the bonds between actin and myosin, in the production of many processed meat products (11, 12). On the other hand, pH decline and other changes in intracellular conditions activate enzymes responsible for tenderization and formation of aroma precursors, as discussed below.

With respect to cultured meat, due to the lack of cultured meat available for scientific study, there is no information available on whether and to which extent such transformations occur (4). Future studies on cultured meat should shed light on glycogen content and pH evolution after harvest to assess the (dis)similarities to traditional meat. Isoforms of actin and myosin in cultured muscles were found to be predominantly neonatal or embryonic, rather than adult (13). This may alter the proteins' response to a potential post-mortem transformation. If these transformations are absent, then muscle is not transformed into meat, which is biochemically dissimilar (14). If *rigor mortis* would be less strong or no actomyosin complex would be formed, this may have a positive effect on the product quality with respect to tenderness and water holding capacity in comparison to traditional meat, while on the other hand, it may change the further aging process.

After slaughtering, meat is aged for tenderization and formation of flavor precursors (15). The aging period depends on the type of meat. In beef, in which a low amount of proteases are present, aging takes ~14 days. The tenderization process is complex, involving many proteolytic enzymes and has been studied for many years but is not entirely elucidated. Calpain, a protease complex present in the sarcoplasm, is thought to play a central role in the process (9). Calpains degrade several myofibrillar proteins, but not actin and myosin (10). Several other enzymes, such as proteasome, caspase (9, 10), or the lysosomal enzyme cathepsins (7, 16) are also involved. The extent to which these enzymes act strongly depends on the microenvironmental conditions, such as pH, ionic strength and oxidative and nitrosylation status of the cell (10). Intracellular conditions in cultured meat may substantially differ from traditional meat, which will influence the rate and extent of tenderization and flavor development.

## Structure and Texture

The technological challenges with respect to cultured meat texture are strongly dependent on the type of meat or meat product that is produced. The challenges to creating an appealing texture in producing cultured meat mimicking fresh meat are by far greater than challenges involved in the preparation of ground or finely minced meat products. It is acknowledged that the production of a full-sized cultured product similar to steak or pork chops is challenging and may not be feasible within the near future (4, 5, 17, 18). Due to the absence of blood, providing nutrients and oxygen, and diffusion limitations, only a few cell layers can be produced using currently available culture techniques (19). The production of thicker meat pieces would require a perfusion system allowing medium with nutrients and oxygen to be distributed throughout the tissue. Assembly of a vascular-like system lined by endothelial cells may be a way to allow such perfusion (20). In traditional meat, the texture depends on the myofibrillar structure as affected by *rigor mortis* and aging, the amount and structure of connective tissue present in the endo-, peri-, and epimysium of the muscle and the amount and composition of fat in the muscle (7, 21). Closely mimicking these properties would require co-culturing of myoblasts with fibroblasts and adipocytes (22). However, co-culture of several cell types is technically challenging, since each cell type grows and differentiates in specific media. When several cell types are cultured in the same medium, these conditions may be sub-optimal for one or more cell types (23). By medium additions, cells can be directed toward increased deposition of extracellular matrix, changing the mechanical properties of the tissue (24). On the other hand, instead of inducing a structure through complex cell co-cultures, a connective tissue structure can also be created by means of an edible (non-cellular) matrix. Such matrix (also called “scaffold”) could be based on connective tissue when made of structural proteins, such as collagen and elastin.

The production of ground cultured meat products, such as hamburgers, is more feasible, as proven by the cultured meat prototype demonstration in 2013 (2). Traditional hamburgers of high quality are produced by grinding meat (beef) using a 3–6 mm blade. The final structure still includes tissue fragments. Binding of these fragments occurs mainly through meat proteins that are extracted by adding a small amount of salt (7). In the patty produced from cultured meat in 2013, 10,000 muscle fiber strips of ~1 mm in diameter were used (1), hence the tissue fragments were significantly smaller. In order to bind these strips and to provide the product with the texture needed, breadcrumbs, egg white powder and binders were needed (18). The texture of the resulting product is therefore expected to resemble industrially processed burgers (which are minced more finely and also contain these additional ingredients), rather than fresh high-quality burgers, which only contain salt as an ingredient.

Other processed meat products, such as cooked sausages, are even more finely minced. In these products, meat is minced to such extent that no cellular structures remain (25). This could reduce the complexity of cultured meat production for this purpose. For example, the use of edible scaffold material for cell culture could be omitted (26). Structure formation in these products strongly relies on the techno-functional properties of the dissolved proteins, more specifically the gelation of the myofibrillar proteins actin and myosin during pasteurization. In addition, if a fat fraction is added (which is the case in cooked sausages), the proteins stabilize the fat by forming an interfacial protein film around the fat globules (7, 25). Hence, the gelling and emulsifying characteristics of meat proteins are of paramount importance in the production of finely minced meat products. It has been suggested that the biochemical composition of cultured meat is expected to be very close to that of regular meat, since both contain muscle fibers (5). However, muscle fibers formed through the currently available *in vitro* methodologies contain only small amounts of predominantly embryonic or neonatal isoforms of actin and myosin (13). Electrical and/or mechanical stimulation increases myofiber diameter, enhances myotube structure and increases myofibrillar protein content (27, 28). It remains to be determined whether such stimulation is scalable, economically feasible and whether the resulting protein content and techno-functional quality would be sufficient to provide the gelling and emulsifying properties needed in the production of such meat products. If not, additional structure forming ingredients would be needed, such as other proteins, hydrocolloids, starches, fibers, etc. Many currently available meat alternatives, commonly based on plant proteins, also contain considerable amounts of structure-forming ingredients in order to correct for their inferior techno-functional properties. However, this addition may lower the product attractiveness to consumers, who demand clean label products.

From a textural point of view, it can be questioned whether entire muscle cells are needed for the production of *in vitro* produced finely minced meat products, as no cellular structures remain after the mincing process (25). The use of synthetic meat proteins produced through fermentation could be a more feasible alternative (29).

## Color

The red color of meat is mainly attributed to the presence of myoglobin, a heme containing protein. Cultured muscle tissues generally have a pale color due to the absence of myoglobin, since myoglobin expression is suppressed at ambient oxygen conditions (18, 20, 30). Several approaches have been suggested to increase the myoglobin content of cultured meat.

A first approach is to increase myoglobin expression by adaption of culturing conditions, for example by culturing muscle fibers under low oxygen conditions (18, 31). However, more research is needed to determine if hypoxic conditions alone are sufficient to increase myoglobin expression (32, 33) and evaluate the impact of low oxygen conditions on the culturing efficiency. Increased glucose consumption and lactic acid production has been reported under hypoxic conditions, suggesting better efficiency (34). However, this may result in medium acidification, which could damage the cells (14, 35). Expression of myoglobin could also be stimulated by presence of media additives, such as lipids or acetic acid (34). In addition to myoglobin protein synthesis, color development also requires the presence of sufficient amounts of iron in the cell. Myoglobin contains heme, which has iron in the center of its structure. Basal media for cell culture contain no iron (e.g., IMDM, RPMI1640) or only a low amount of iron in the form of ferric nitrate non-ahydrate (DMEM: 0.1 mg/L) or ferrous sulfate heptahydrate (Ham's media 0.8 mg/L). Supplementation of the cell culture medium with extra iron results in an increase of iron content of the cells, although only part of the iron is taken up, suggesting there might be a limit to the amount of nutrients the cells can incorporate (36). Uptake is dependent on transferrin, a protein which binds iron and mediates transport in the cell (34, 35). The extent to which iron is then incorporated into heme (necessary for good iron bio-accessibility) and myoglobin (necessary for color development) remains to be studied (18).

A second approach to increase the myoglobin content in cultured cells is the direct addition of myoglobin to the medium. In a recent study by Simsa et al. (31), the addition of metmyoglobin was shown to increase the cell proliferation capacity and resulted in an increased myoglobin content in the cultured cells. However, myoglobin contents were still much lower compared to beef, and the resulting color was brown, resembling cooked beef rather than fresh beef which was due to the use of metmyoglobin (the oxidized form of myoglobin).

Failure to incorporate sufficient amounts of myoglobin in the cultured cells would necessitate the external addition of myoglobin or other colorants at a later stage in the production process. This would only be possible for processed meat products. In this regard, an artificial colorant, soy leghemoglobin, produced via a genetically engineered *Pichia pastoris* (37), recently obtained FDA approval for incorporation in a plant-based burger, giving it the color and taste of a natural beef burger (38). However, it is not clear whether soy leghemoglobin could be applied in the context of cultured fresh meat. Finally, it must be noted that red meat has been associated with increased incidence of several types of cancer (39). While the exact mechanisms are not completely understood, the potential role of heme iron has been pointed out in this respect (39). Therefore, from a health perspective, the use of alternative colorants instead of heme might be pursued for cultured meat.

## Flavor

Fresh, uncooked meat has little flavor. It tastes rather bloody (15, 40), which is attributed to its relatively high iron content. As discussed in the previous section, the iron content in the cells can be increased to some extent by using iron-fortified medium. Other compounds contributing to the taste are lactate (sour taste) and inosine 5′-monophosphate (IMP, umami taste), both formed during post-mortem metabolism (15). Upon heating, complex thermally-induced reactions result in the formation of enormous numbers of volatiles, some of which (but not all) contribute to the typical meat flavor. The main reactions involved are the Maillard reaction and lipid degradation reactions, as well as interactions between both (15, 40).

Maillard reaction involves a reaction between an amino compound (free amino acids or peptides) and a reducing sugar (mainly ribose and ribose 5′-phosphate, which are a breakdown products of IMP). In traditional meat, substantial amounts of these precursors are formed during post-mortem metabolism. It is unclear to what extent these flavor precursors will be present in cultured meat, in which the prevalence of post-mortem metabolism has not been studied due to the lack of cultured meat currently available.

Lipid degradation upon cooking occurs even in very lean meat and meat products, due to the presence of intracellular lipids and especially phospholipids from membranes, which generally contain a higher amount of polyunsaturated fatty acids that are more susceptible to oxidation (15, 40). When higher amounts of fat are present, the contribution of these volatiles to the overall flavor increases (40). While oxidation products contribute to the desirable aroma of meat, they can also cause off-flavors (e.g., warmed-over-flavor) and are often the cause of meat spoilage (15). When considering the presence of fat in cultured meat, again the distinction between fresh meat and processed meat is necessary. On the one hand, in fresh meat, fat is known to contribute significantly to the taste of the product, as well as the texture and juiciness. Adding a fat fraction to cultured meat may require co-culture of muscle cells with adipocytes. On the other hand, in finely minced meat products, fat (in most cases pork back fat) is often added as a separate raw material (7). Analogously in cultured meat, fat may be added at the end of the culture process and alternatives, such as (separately) cultured fat or plant-based fat may be used instead of animal-derived fat. From a technological point of view, the addition of alternative fats in finely minced meat products is well-described (41, 42).

In case the culturing process itself does not result in a product with satisfactory flavor, addition of artificial flavor compounds akin to those currently used in plant-based meat substitutes (22) might be an option.

It can be added that in cultured meat, some specific problems related to off-flavors that occur in some traditional meats can be avoided. An example is boar taint, an off-odor present in uncastrated male pigs, related to the presence of androstenone, indol, and skatol (43).

## Nutritional Composition

Meat is generally considered as a nutritious product due to the presence of highly digestible proteins with excellent amino acid composition, vitamins, and minerals. With regard to proteins, some considerations have already been given in section Structure and Texture. It is not clear to what extent the protein content and composition of cultured cells resembles that of traditional meat.

Scaffolds composed of naturally occurring polymers are commonly used as a way to organize cells in a 3D environment (14). In current tissue engineering approaches, a hydrogel of such polymers is often used as this facilitates cell-induced contraction and tissue alignment. The hydrogel volume typically largely exceeds that of the cells, even after prolonged culture time (27); therefore macronutrient composition of the overall product will also be affected by the scaffold material. Proteins, such as collagen or fibrin are already used in muscle tissue engineering approaches. Collagen contains mainly non-essential amino acids (44) but also a moderate amount of lysine, which is considered a limiting amino acid in diets devoid of meat (45). However, lysine in collagen of connective tissue is to a varying degree post-transcriptionally modified to hydroxylysine which cannot be used in protein synthesis (46). Therefore, it will be of interest to determine the amount of lysine vs. hydroxylysine in the collagen, which will be dependent on the source (different types of animal collagen or recombinant collagen). In lean meat, collagen makes up only a small fraction, but in the case of processed meat products, it can be added to constitute up to 25% of total protein (47). To avoid animal-derived components, polysaccharides, such as alginate, cellulose or chitosan (derived from algae, plants, and fungi, respectively) could be used as scaffold material, providing a source of dietary fiber, which has numerous health benefits and is underrepresented in western diets (48).

From nutritional point of view, fat in meat can be characterized by its percentual content and fatty acid composition. These characteristics are influenced by variables, such as livestock species and breed, age, type of feed, and meat cut (49). While overall fat content impacts mainly the caloric density of the product, fatty acid composition influences the dietary value in more complex ways (saturated or unsaturated fat, ratio of polyunsaturated fatty acids, trans-unsaturated fats). Addition of fatty acids can be pursued by co-cultures of adipocytes derived from adipose stem cells, which can synthesize various saturated and unsaturated fatty acids (50). However, essential fatty acids (mostly linoleic and α-linolenic acid) and some other nutritionally valuable compounds (e.g., conjugated linoleic acid, synthesis of which depends on biohydrogenation occurring in ruminants) present in meat (49) may still be missing in the co-culture approach. More research is needed to determine whether the fatty acid composition of adipocyte culture can be manipulated for instance by directly adding essential fatty acids to the media without disrupting growth and lipogenesis (51). Alternatively, end-stage addition of (plant-based) fats in cultured meat products may be economically and technically more feasible compared to *in vitro* co-culture with adipocytes.

Meat is also a significant dietary source of minerals, such as iron, zinc, and selenium. In muscle tissue, iron is either present as a part of a heme group in myoglobin (and to lesser extent hemoglobin) or stored in complex with ferritin in a non-heme form (52). From a nutritional standpoint, it is advantageous to consume iron in the heme form, because it is absorbed more easily than the non-heme form and its absorption is not hindered by chelating agents naturally occurring in some foods (53). Increasing myoglobin content would therefore improve nutritional characteristics in addition to color and taste properties. Other minerals, such as zinc and selenium are either not present in basal cell culture media (e.g., DMEM, RPMI1640) or in very low concentrations (Ham's media contain zinc sulfate heptahydrate and IMDM contains sodium selenite) and thus need to be supplemented to support cell growth. Thus far, nothing is known about the uptake of these minerals in cultured meat.

In most diets, meat provides a large share of various B-group vitamins, especially B12 (8). The latter vitamin is synthesized exclusively by microorganisms (bacteria and archea) and then absorbed and utilized by animals, while plants rarely contain considerable amounts of B12 (54). Hence, people following plant-based diets need to take vitamin B12 supplements in order to fulfill their dietary demands (55). If cultured meat is to be regarded as a substitute for traditional meat, it is vital that it contains vitamin B12. With regard to tissue engineering, vitamins are necessary in the media for optimal cell proliferation (56), but it is not clear whether the uptake from media results in levels of vitamins in cultured meat comparable to traditional meat. Furthermore, uptake of B12 requires a binding protein (transcobalamin II) enabling transport across the cell membrane (55, 57, 58). This can potentially present an additional challenge to achieving adequate levels of B12 in cultured muscle tissue. Further research is needed to determine if spontaneous vitamin uptake mechanisms are sufficient to achieve nutritional parity with traditional meat. An alternative approach would be the post-culture addition of vitamin B12 to the meat (product). Similarly, many currently available plant-based meat alternatives contain added vitamin B12 in order to enhance their nutritional value.

Aside from crucial nutrients, such as vitamins, minerals, and essential amino and fatty acids, meat also contains numerous bioactive compounds beneficial to human health. Taurine is a free amino acid playing a vital role in many metabolic processes (59). In humans, it is partially obtained from diet, but internal synthesis of taurine, occurring mainly in liver and brain, is sufficient in healthy humans (60). However, high dietary intake has been associated with a protective effect against cardiovascular diseases (61), and increasing the taurine content of cultured meat might therefore be beneficial. Furthermore, the potential of cultured meat as an ingredient in pet food is currently being explored (62), since pet food creates 25–30% of the total environmental impact from animal production in the US (63). Taurine is an essential nutrient in cats and conditionally essential in dogs (64), making taurine addition necessary for this application, considering general cell culture conditions are taurine deficient. Taurine treatment enhances the differentiation of myoblasts to myotubes (65), therefore addition of taurine to the cell culture media may increase efficiency of the production process, in addition to its nutritional benefits.

Creatine, a substance widely known to accumulate in muscle where it provides an instantaneous source of energy for contraction, is synthesized mainly in liver, kidney and pancreas. Dietary supplementation has been extensively studied and found to be beneficial for gain of muscle mass and to a certain extent also improvement in cognitive function in healthy adults and the elderly (66). Moreover, addition of creatine to the cell culture media improves myoblast differentiation (67) and could therefore be used to improve cultured meat production. However, increasing the creatine content might also have an accidental adverse health effect. As a result of the Maillard reaction during cooking, creatine in traditional meat forms carcinogenic heterocyclic amines (68). Other compounds in traditional meat products, such as N-nitroso compounds and heme iron have also been associated with increased cancer risk (39). It remains to be seen whether the levels of these compounds could be lowered in cultured meat without compromising sensorial and nutritional aspects.

## Conclusion and Future Challenges

Due to technological challenges related to its production, cultured meat prototypes are currently not available for independent technological, sensorial and nutritional assessment. Based on the available state of the art regarding production processes, it can be inferred that cultured meat currently differs significantly from traditional meat in its technological, sensorial and nutritional properties. Revealing the extent to which post-mortem processes occur in cultured meat is crucial to understand its impact on sensorial and technological properties. Production of cultured meat resembling fresh, unprocessed meat entails the biggest challenges with respect to texture, color, flavor as well as nutritional composition. Ideally, this would entail co-culturing of myoblasts with fibroblasts and adipocytes. In addition, electrical and/or mechanical stimulation may be needed to improve the techno-functional quality of the meat proteins. However, the technological and economic feasibility of these solutions, especially at large scale, can be questioned (13). With regard to nutritional value, we illustrated the long trajectory of additional research that is needed before the composition of cultured meat could resemble traditional meat, as well as the complexity of the medium composition needed to achieve this. This will not only add to the cost of the medium, but also increase the environmental footprint of the entire process. In processed meat products, most of the challenges mentioned above may be overcome by the simple addition of texturizing ingredients, colorants, flavorings and nutrients in order to remedy the sensorial and nutritional properties. However, this decreases consumer acceptability. Further, in the absence of a defined and openly communicated production process, it is currently impossible to gauge all potential issues related to sensorial aspects and nutritional value of cultured meat products entering the market in the forthcoming years.

## Author Contributions

IF initiated the sections on structure, texture, color, and flavor from the perspective of a meat technologist. MK initiated the section on nutritional composition. LT and HV added the information throughout the manuscript relating to muscle tissue engineering and edited in all sections. All authors had contributions throughout all sections, read, and approved the final manuscript.

### Conflict of Interest

The authors declare that the research was conducted in the absence of any commercial or financial relationships that could be construed as a potential conflict of interest.
